# Technology for Fundamental Improvement of an Extremely Low-Quality Video Signal for Use in Fine Focusing and Astigmatism Correction in Scanning Electron Microscopy

**DOI:** 10.1155/2023/7305255

**Published:** 2023-01-25

**Authors:** Eisaku Oho, Sadao Yamazaki, Kazuhiko Suzuki

**Affiliations:** ^1^Department of Electrical and Electronic Engineering, Faculty of Engineering, Kogakuin University, 2665-1 Nakano-machi, Hachioji, Tokyo 192-0015, Japan; ^2^Research & Development Center, Nohmi Bosai Ltd., 1-18-13, Chuo, Misato, Saitama 341-0038, Japan

## Abstract

This study describes important techniques for production of a series of video signals for use in fine focusing operations and near-perfect astigmatism correction in the general-purpose scanning electron microscopy (SEM) field. These techniques can enhance the stability of the signal greatly when used for focusing. As two particularly important fundamental techniques, SEM image acquisition with priority given to the signal-to-noise ratio and signal reinforcement based on the active image processing concept were utilized fully. The performance improvement was evaluated using the case of a previously reported support system for fine focusing and astigmatism correction based on image covariance. The method is almost completely robust against noise within practical limits and allows for focusing and astigmatism correction for even extremely noisy SEM images. The results of this study may be useful not only in the SEM field but also in many fields that use weak signals.

## 1. Introduction

Many scientific instruments require focusing. However, both manual and automatic focusing operations may be affected by noise. In particular, in the high-magnification and high-resolution observations made in general-purpose scanning electron microscopy (SEM), which uses an electron beam current on the picoampere scale, the focusing performance often varies with the quality of the SEM signal, which may include severe noise. Field emission guns have been used to improve the signal-to-noise ratio (SNR) of SEM images dramatically. However, under both low- and high-magnification conditions, the problem has yet to be resolved because the image quality is strongly dependent on both the characteristics of the specific specimen (e.g., surface structural details that can only produce very weak image contrast) and the SEM operating conditions.

During SEM image observation, after image acquisition, and during the adjustment of the various SEM parameters performed prior to image acquisition (e.g., focusing), digital image processing techniques with a statistical backing based on the SEM image characteristics should be used to reduce the adverse effects of noise and/or blur substantially. In the early years of study in this field, many techniques to improve the SEM image quality were simply adopted from the image processing field [1]. At present, useful results from several of these studies are being used in practice in commercialized and/or prototype SEMs. For example, as practical methods for use during SEM image observation, a recursive filtering method (involving averaging over multiple TV-scan SEM images), was introduced to reduce SEM noise [2], and an image restoration system (to reduce blur) was developed for TV-scan moving images that were acquired using a semiconductor backscattered electron detector [3]. In addition, modern image restoration methods based on estimation of the beam intensity distribution have been proposed to improve the resolution of previously acquired SEM images [4, 5]. Another method was proposed to produce a denoising result that was basically equal to the resolution of the acquired SEM image (for preservation of fine structures) based on evaluation of the noise amplitude of the SEM image [6].

Recently, a denoising method for critical dimension (CD) SEM images based on deep learning was developed [7]. Because this method does not require to be trained with clean ground-truth images, it will be highly effective for use in the SEM field (it is very difficult to obtain large numbers of SEM images with extremely low noise). However, because we would have to use this method at very high processing speeds in the focusing operations in this study under a variety of conditions, including different magnifications, microstructure geometries, degrees of noise amplitude, and degrees of image blurring, it is difficult to adopt it at present. In another very recent study, a deep learning-based refocusing method for blurred SEM images was proposed (an image restoration method to improve the resolution of previously acquired SEM images) [8]. The results for this method seem to have been obtained under the condition that very little SEM noise was present. In addition, only simple image blurring was considered, and the adverse effects of image distortion caused by astigmatism have not been discussed to date. Therefore, this concept was also unsuitable for use in this study.

Several methods, including techniques that use Fourier transforms [9–14], autocorrelation functions [15], and image variance [16] have been used to perform (automatic) focusing of an electron beam [11, 15, 16] and to evaluate sharpness (i.e., image quality) [9, 10, 12–14] in SEM applications, which are the aims of this study. Unsurprisingly, these methods will also be strongly affected by noise. Erasmus and Smith [16] and Oho et al. [17] both used covariance to achieve more precise focusing under noisy conditions. As an advance from this study, the SNR of an SEM image with the same origin as the covariance is adopted as an easy-to-use index for focusing evaluation, because the measurement of the SNR does not typically vary under the same focusing conditions, even if the contrast and brightness are both adjusted freely [18]. In support of this approach, a comprehensive comparison study reported that the normalized variance, which has the same origin as the covariance, is the best index for use in focus evaluation among the many indexes available for noisy optical microscope images [19]. However, despite many research findings to date, fine focusing operations and near-perfect astigmatism correction for SEM remain difficult tasks under extremely noisy conditions.

To solve the difficult problems described above by improving the signal quality to be used for fine focusing and astigmatism correction, it is helpful to devise an appropriate combination of the image processing and SEM technologies required. If these improvements can be made, optimal SEM performance will be possible under noisier conditions. In this study, we use both an SEM image acquisition method with priority given to the SNR [17] and the active image processing concept [20, 21] to provide a suitable solution. The former method maximizes specific image information required for fine focusing, while the latter prioritizes the development of various SEM functions to acquire SEM signals that include sufficient information. Both of these simple technologies are central to improved acquisition and processing of the focus detection signal. We demonstrate that the proposed solution can clearly be effective under poor SEM conditions that have not been discussed previously.

## 2. Experimental System to Improve Signal Quality during Focusing

**Figure 1:**
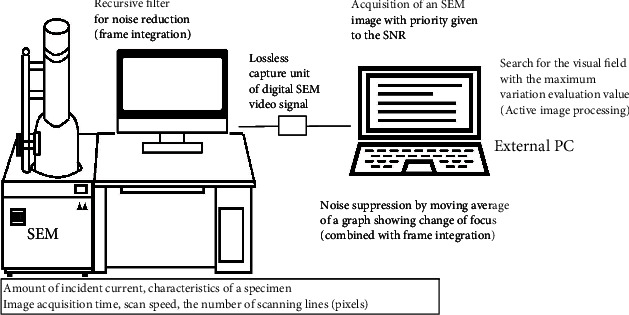
Experimental system for signal quality improvement during focusing. Numerous ideas to improve the quality of the SEM video signals used for fine focusing and astigmatism correction are available.

Figure 1 outlines the system used to improve an extremely low-quality SEM video signal for use in focusing. Digital SEM signals that are output from a Hitachi S-3400N (general-purpose SEM with a thermal electron emission-type electron source composed of a tungsten hair pin; Hitachi High Technologies, Tokyo, Japan) are typically used in the signal improvement system presented here. We use a TV scan (in this case, 25 frames/s) in this study, except in specified cases. The SEM digital video signals are acquired continuously using a personal computer controlled via LabVIEW software (National Instruments, Austin, TX). To obtain better imaging results, the personal computer is equipped with a DVI3USB 3.0 video grabber (Epiphan Systems Inc., California, USA) for lossless video capture from a device with a digital visual interface output port.

**Figure 2:**
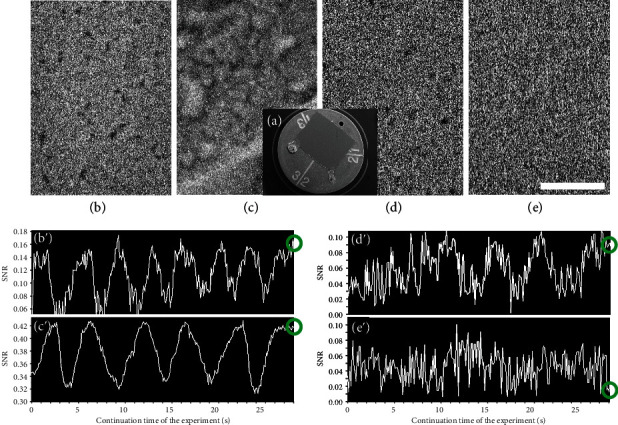
Confirmation of the quality of the just-focused images and graphs for focusing evaluation in the case without use of noise reduction technologies. (a) Flat specimen and its specimen stub, which were coated with a thick Au film. (b) SEM image of the flat thick film acquired at 26 pA. (c) Image of the specimen stub acquired at 26 pA. (d) Image of the film acquired at 8 pA. (e) Image of the film acquired at 2 pA. (b′–e′) Graphs showing the changes in the SNR during the focusing operation corresponding to each condition. The bar represents 0.7 *μ*m.

**Figure 3:**
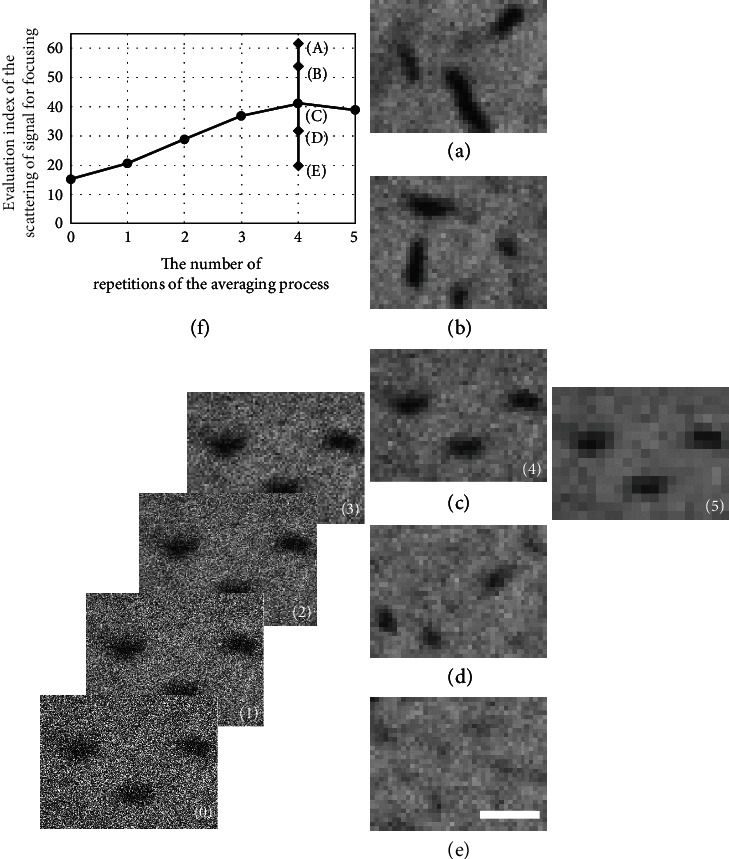
Improvement in the signal for focusing operations produced by moving the electron beam. To improve the desired signal within the weak SEM signal fundamentally, the field of view with the maximum variation evaluation value is located near the observation region. (a–e) Search candidates (with 300,000x magnification). (a) is used to improve the signal for focusing operations. (f) Points a–e show the changes in the variation evaluation value. For an overall view, all results from (0)–(5) obtained during the process of creation of (c) when using the SEM image for which priority is given to the SNR are shown. See text for full details. The bar represents 0.14 *μ*m.

As Figure 1 already shows, many ideas have been proposed to improve the quality of the SEM video signals used for fine focusing and astigmatism correction applications. It is possible to change the image quality (particularly the SNR) by adjusting several of the SEM parameters within the rectangular frame. However, because it is difficult to increase the incident current for general-purpose SEM without degrading the resolution and because a fairly fast scan is required for focusing operations, we cannot often expect the SEM signals to have a high SNR. In addition, the SNR variations are strongly dependent on the properties of the specimen under observation (e.g., the features of fine surface structures). These typical examples will be confirmed experimentally later (see Figures 2 and 3). With these considerations in mind, we will now discuss the important aspects of image processing techniques (outside the rectangular frame shown in Figure 1) that can be used widely and effectively to improve signal quality for fine focusing.

### 2.1. Acquisition of an SEM Image with Priority Given to the SNR

An SEM image is acquired for a predetermined number of scanning lines (pixels) and a predetermined acquisition time, which are both correlated strongly to the SNR of the acquired image. If the acquisition time remains constant, we can then place greater importance on either the SNR or the number of pixels used *M* via appropriate setting of the SEM parameters. To consider which of these aspects is more important in the focusing work, we use the following equations [16, 17, 22]:

(1) $$\begin{array}{c} \frac{E\left\{\text{Co}{\text{v}}_{s}\left(t_{1},t_{2}\right)\right\}}{\sqrt{\text{Var}\left(\left(\text{Co}{\text{v}}_{s}\left(t_{1},t_{2}\right)\right)\right.}}=\sqrt{\text{number of pixels }M}\frac{\text{SN}{\text{R}}^{2}}{\sqrt{2\text{SN}{\text{R}}^{2}+1}}, \end{array}$$

(2) $$\begin{array}{c} \text{SNR}=\frac{S_{\sigma }}{N_{\sigma }}=\sqrt{\frac{\text{Cov}\left(t_{1},t_{2}\right)\;}{\sqrt{\text{Var}\left(t_{1}\right)\cdot \text{Var}\left(t_{2}\right)}-\text{Cov}\left(t_{1},t_{2}\right)}}, \end{array}$$

where *E*{} represents the statistical expectation. Close examination of Equation (1) indicates the potential usefulness of SEM noise immunity. This important feature would be helpful in focusing operations. The numerator and denominator on the left-hand side of Equation (1) show the desired signal (squared) and the standard deviation of the aggregation of multiple sample covariance (Cov_*s*_) values, respectively. In other words, the left-hand side represents an index of the theoretical scattering of the measurements of the covariance in the case where a sample has *M* pixels. A higher value of this index will yield a better (i.e., stable) covariance measurement. Next, the right side is derived from the left side of Equation (1) and is composed of the SNR and *M*. Here, the SEM signal includes the desired signal *S*_*σ*_ and the noise *N*_*σ*_, and the SNR is defined as the ratio of the standard deviation of *S*_*σ*_ to the standard deviation of *N*_*σ*_. For the measurement of SNR as shown in Equation (2), we require two images (*t*_1_, *t*_2_) taken from the same viewpoint, and their covariance (Cov(*t*_1_, *t*_2_)) and individual variances (Var(*t*_1_) and Var(*t*_2_)) are then calculated. *S*_*σ*_ is equivalent to the square root of the covariance value above. The reason for there being no subscript *s* for the covariance (Cov) shown in Equation (2) is that the equation expresses the true covariance rather than the sample covariance. The right side shows the scattering of the sample covariance measurements expressed using only the SNR and *M* and indicates that it is possible to adjust the SNR and *M* suitably under specific conditions to improve the numerical value of the index. The right side of Equation (1) clearly shows that stable measurement of the covariance in our study can be achieved by improving the SNR rather than increasing *M*. We must therefore select an SEM image acquisition method that increases the SNR as far as possible.

**Figure 4:**
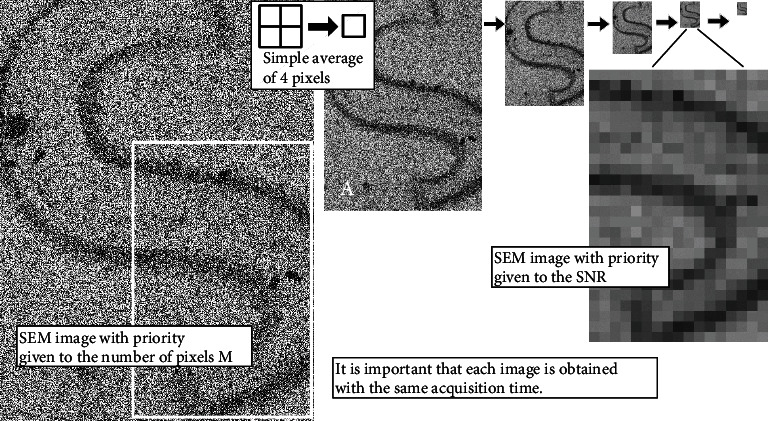
Advantage of SEM image with priority given to the SNR for use in focusing operations and a procedure to obtain the desired processed image. See text for details.

**Figure 5:**
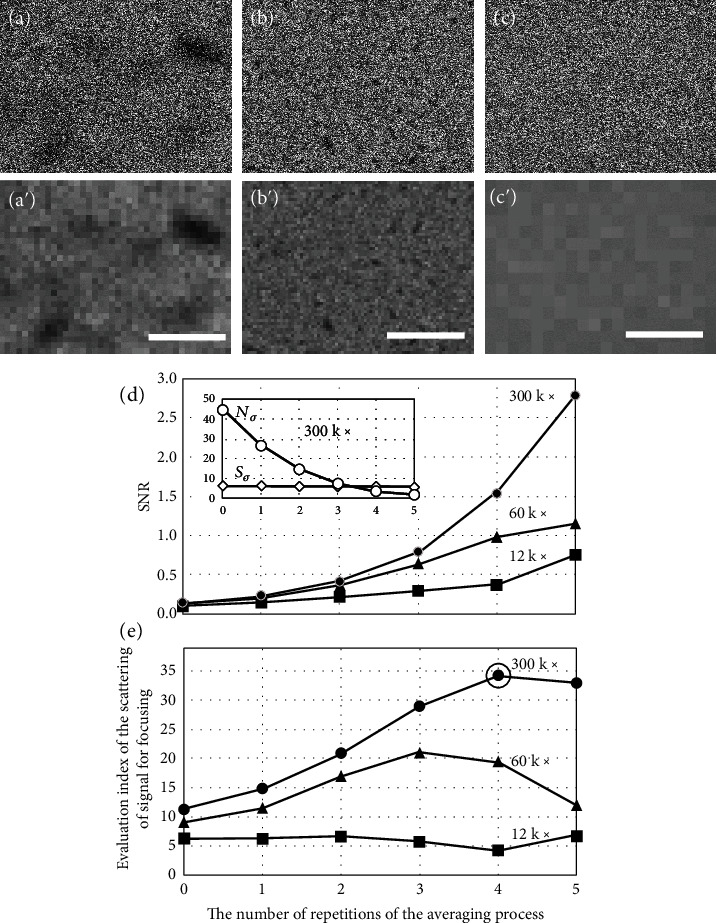
Performance confirmation of the SEM image acquisition method when priority is placed on the SNR. To determine a suitable magnification condition for this method and for our SEM instrument, three magnifications ((a) 300,000x, (b) 60,000x, and (c) 12,000x) were selected for the experiments. The images were processed using the procedure described in Figure 4, and the changes in their SNRs with repetition of the averaging operations for 2 × 2 pixels are plotted in (d). (a′)–(c′) show the processed results for the SEM images acquired at each magnification. Next, an evaluation index of the scattering of the signal for focusing operations from Equation (1) was obtained as shown in (e), and the maximum value (as identified by a small circle) of all the calculated values was selected for our purpose. See the main text for details. The bars represent 0.14 *μ*m (a and a′), 0.7 *μ*m (b and b′), and 3.5 *μ*m (c and c′).

Figure 4 presents a procedure to acquire an SEM image while prioritizing the image SNR. For example, if the number of pixels decreases by a factor of four, the standard deviation (amplitude) of the SEM noise is then theoretically halved. As a result, the SNR increases by a factor of 2. After image acquisition, the reduced image (indicated by A) can be realized by simple averaging over 2 × 2 pixels, although this reduces the number of pixels used. A comparison of the case where this process is repeated four times is illustrated visually in Figure 4. When compared with the (unprocessed) SEM image in which priority was given to the number of pixels used (at the left side of Figure 4), although the number of pixels has decreased dramatically, the SNR has improved sufficiently in the SEM image in which priority is given to the SNR (on the right side, with the image expanded to enable comparison). This method is similar to the application of a low-pass filter, but the result (i.e., the improved SNR) can be handled successfully in Equation (1) in combination with the change in the number of pixels *M*. This use of an increase in the SNR rather than a reduction in the number of pixels is particularly effective for data that have been acquired under very low SNR conditions, and it is helpful for focusing operations. Indeed, SEM instruments have long been equipped with reduced images that are available to the user for focusing. However, assuming that these SEM images can be reduced in size freely, the method required to obtain the best possible results has probably never been discussed on the basis of sufficient available experimental results. Within permissible limits, the original SEM image should be reduced in size to improve the SNR. In other words, if the desired signal more than satisfies the sampling theorem (e.g., in the case of unusually high magnification in general-purpose SEM), then the averaging approach in question can be used to provide nearly ideal improvement in the SNR (i.e., where only the noise component is reduced). As a result, as indicated by the relationship between the SNR and the number of pixels *M* in Equation (1), the variations in the measurements of the sample covariance will be reduced dramatically. These typical examples will be confirmed experimentally later in the paper (see Figure 5). In this study, the SNR itself and the covariance are used to form the index for focusing operations. The scattering of the SNR measurements obtained from images with *M* pixels is similar to that of the sample covariance, because the denominator in Equation (2) usually remains almost constant during focusing operations.

### 2.2. Reinforcement of the Signal for Focusing Based on the Active Image Processing Concept

It is obviously important to acquire desired signals that are as strong as possible to enable successful fine focusing and astigmatism correction processes under very low image quality conditions. However, most obvious techniques to improve the signal quality through image processing and setting of the SEM parameters have already been examined, including those in the discussion above. We therefore focus on another important measurement aspect, i.e., the specimen itself, which has not been addressed to date for signal reinforcement in focusing operations. The SEM image quality is related to the size, shape, and composition of the fine details of the specimen under study. In this work in particular, because unusually high magnification is used in the general-purpose SEM (with a thermal electron emission-type electron source composed of a tungsten hair pin), the desired signal included in the SEM image is strongly dependent on the states of the surface structures included within the visual field (as will be discussed in later sections). Under these circumstances, we use the active image processing concept [20, 21] to improve the SEM signal.

The concept of active image processing can be easily explained using the example of an X-ray computed tomography (CT) scanner commonly used in hospitals. The equipment has a large number of detectors set in advance, and sufficient information for the image processing is obtained during signal acquisition. Therefore, when subsequently reconstructing a tomographic image of an organ or structure in the body, the work load of the image processing technology is low, and it is expected that the resulting image is always of high quality and stable. Here, the CT scanner is able to operate as a high-performance instrument because the data (image) processing method and data acquisition method discussed below are developed to cooperate with one another, which is the concept of active image processing. If the number of detectors is insufficient, excessive demand is placed on the image processing to compensate for the missing information. Unfortunately, the quality of the tomographic images and other images obtained will often be unsatisfactory.

With the above concept in mind, in the following, we will discuss active image processing adopted in the focusing operation of SEM images. The purpose of the image processing is first explained, and a data (image) processing method and a data acquisition method that are suitable for that purpose are then developed together. Consequently, there is a high possibility that the goal of active image processing described in Step 1 can be achieved because it is anticipated that SEM images that contain sufficient information for data processing will always be available.

**Step 1 .**

The purpose of the active image processing is clarified.

Fine-focusing operations under a high-noise condition are nearly perfectly enabled by improving the quality of the SEM signal to be used for focusing.

**Step 2 .**

The data processing method and data acquisition method are considered together for the above purpose.

#### 2.2.1. Data Processing Method

The SNR value is usually selected as the signal for focusing, i.e., the focus evaluation index value, because freely adjusting the contrast and brightness of the SEM image does not change the measured SNR. Here, the performance of the covariance method, which is the most important part of the process of obtaining SNR values (i.e., the degree of scattering of the measurements of the covariance evaluated in the previous section), should be evaluated in advance. Meanwhile, it may be better to use the covariance itself when manufacturing an automatic focus adjustment device using the results of this research, from the viewpoint of reducing the computational cost.

#### 2.2.2. Data Acquisition Method

**Figure 6:**
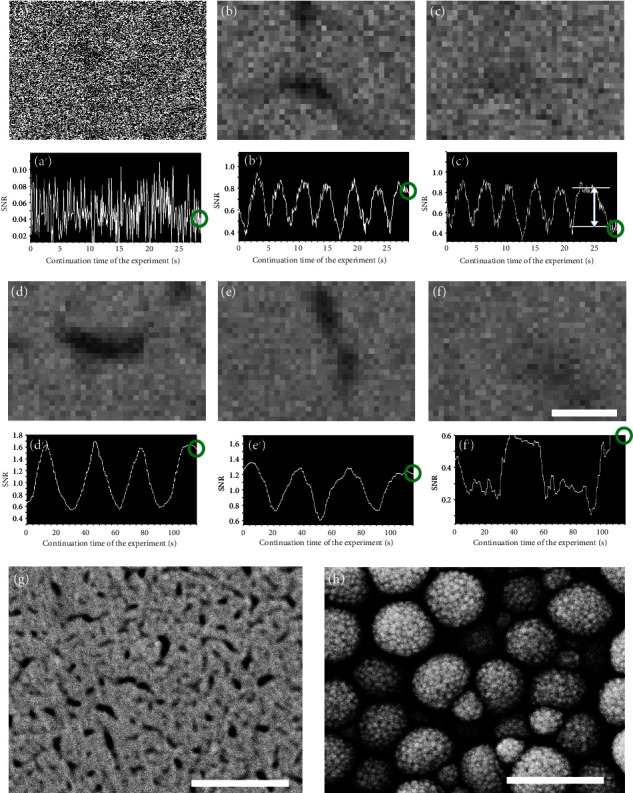
Test of system robustness against noise during fine focusing operation for extremely noisy SEM images. (a) Unprocessed TV-scan image acquired at 3 pA (300,000x). The graph of (a′) does not work at all. (b) The image processed (focused) using the procedure described in Figure 5(b′) works well, as expected. (c) Processed image (defocused) of the identical view. The graphs in (b′) and (c′) are continuous. To provide further stabilization, the moving average (average of four points on a graph for the focusing operation) and the frame integration (integration of four TV-scan images) were also used for (d) and (d′) (fine focusing) and for (e) and (e′) (astigmatism correction). (f) and (f′) show the results that confirm the validity of the method used in Figure 3. (g) Slow scan image. (h) Additional slow scan image of Au-coated silica particles. See text for details. The bars represent 0.14 *μ*m (a–f), 0.7 *μ*m (g), and 0.42 *μ*m (h).

The quality and quantity of data required for the proposed data processing method must be estimated in advance. We will develop a more effective data acquisition method if it is found that the data are insufficient. In this study, the field of view with the highest SNR, corresponding to the strongest desired signal within the weak SEM signal, is found to be located near the observation region, primarily by shifting the electron beam. A rough focus is required in advance to use this function effectively (see Figures 3 and 6).

**Step 3 .**

It must be confirmed whether the acquired data are sufficient (i.e., whether the scatter of the measured covariance values evaluated in the previous section is within a suitable range in the specimen to be observed). In the case that further improvement is required, expansion of the search area is considered to result in the acquisition of a stronger SEM signal. The scanning speed, number of pixels, frame integration numbers of the images, and incident current have already been examined as technical elements of the SEM.

**Step 4 .**

Operations are performed for fine focusing and astigmatism correction.

Using the proposed data acquisition method, sufficient data should be obtained. It is thus highly likely that the focusing operations will be successful. In particular, the active image processing concept is useful in SEM when using image signals of unknown quality from the unknown fine structures of a specimen in a focusing operation.

In contrast to the active image processing case, when general passive image processing is conducted, an SEM image is first acquired in a conventional manner (i.e., without considering the sample characteristics and/or the instrument parameters), and then, the need for noise suppression arises, and after the investigation of the properties of the acquired image, we consider using various applicable smoothing techniques. However, most of these techniques often require fairly complex parameters that differ from image to image and that are dependent on the varying visual perceptions of the operators for appropriate use. In other words, SEM images that are acquired using different SEM parameter values (e.g., values of the magnification, the acquisition time, and the incident current) are disturbed to greater or lesser degrees by use of these techniques because of unsuccessful setting of the processing parameter values. It is difficult to predict these adverse effects. In addition, it is almost impossible to increase the amount of information included in a simply and passively acquired image through image processing alone. For these reasons, the simple application of passive techniques (e.g., low-pass filtering and unsharp masking) to certain low-quality SEM images will not be successful.

### 2.3. Confirmation of the Quality of Just-Focused Images and Graphs for Focusing Evaluation in the Case without Noise Reduction Technologies

Some important experimental results that are relevant to the previous discussion are described in this section. First, we confirmed the quality of the just-focused TV-scan images and the graphs of the changes in SNR used for focus evaluation when the noise reduction technologies in question were not used. An experiment was conducted at an operating voltage of 15 kV, with a working distance of 4.1 mm, dimensions of 640 × 480 pixels, and magnification of 60,000x.

A thick Au film was sputtered on a glass substrate, and this specimen was observed under various incident currents, where a small but deep hole was used as a substitute for a Faraday cup (Figure 2(a)). Figures 2(b) and 2(c) show SEM images of the thick film (black small structures) and the specimen stub itself, respectively, acquired at an electron beam current of 26 pA (the fine structures on this thick film do not produce very strong image contrast, but they are suitable for use in this study for discussion of noise problems). These images are noisy because they are TV-scan-type images (with an acquisition time of 0.04 s). In addition, a fairly small current was used to obtain high image resolution. Figure 2(b′) shows the corresponding graph when the focus knob is adjusted manually. The vertical and horizontal axes of the graph are the SNR and the continuation time of the experiment, respectively. The SEM is determined to be in focus when the SNR reaches one of its highest values. The small variations in the graph shown in Figure 2(b′) that are spread throughout the signal used for the focusing operation may be largely caused by the SEM noise included in the electron beam. Figure 2(b) was acquired under the just-focused condition identified by the small circle at the right end of the graph in Figure 2(b′). However, the system's ability to locate the change from an increasing gradient to a decreasing gradient on the graph (i.e., the focusing point) was affected somewhat by the noise. This indicates the fundamental performance limitation of the focusing evaluation system developed previously by our group using the covariance and the SNR in the cases where the noise suppression techniques were not used [18]. In contrast, these small variations were suppressed more strongly in Figures 2(c) and 2(c′) because the SEM signal acquired from the sample was strengthened by the more intense unevenness, even though the incident current remained the same. Here, the SNR improved by almost a factor of three. It was noted that the SNR was affected strongly by the differences in the fine structures within the observation field of view, even for samples with the same coating thickness. This will be very important for the experimental results that are described later in the paper.

Figures 2(d) and 2(e) show SEM images of the thick film acquired at the currents of 8 and 2 pA (lower incident currents), respectively. It may be possible to locate the best focal position in Figure 2(d′) by luck, but this is not possible for Figure 2(e′) because of the extremely noisy image (Figure 2(e)) acquired with the poor SNR. This SNR would be too low to enable correct measurement [23].

### 2.4. Performance Confirmation of the Method SEM Image Acquisition with Priority Placed on the SNR

Now that the necessity of this study has been demonstrated experimentally, we will attempt to stabilize the focusing operation by using an SEM image that was acquired with priority given to the SNR, as described earlier. To determine the most suitable magnification condition for this method and for our SEM instrument, three magnifications (300,000x, 60,000x, and 12,000x) were selected for the experiment. It should be noted that 300,000x is an excessively high magnification for our SEM, and it would not be sensible to use this level of magnification under normal circumstances. The respective SEM images are shown in Figures 5(a)–5(c) (during acquisition of these images, the position of the specimen remains almost the same). Because the SEM operating conditions and the specimen are the same as those used for the measurements in Figure 2(b) (26 pA), there are likely to be no serious adverse effects from noise in the important data in Figure 5. These images are processed according to the procedure illustrated in Figure 4, and the changes in their SNRs with repetition of the averaging operation for 2 × 2 pixels are plotted in Figure 5(d). Here, the vertical and horizontal axes are the SNR after the averaging operation and the number of averaging process repetitions, respectively. In the 300,000x magnification case, the SNR increases more rapidly than in the other cases because the desired signal *S*_*σ*_ is hardly reduced by the averaging process (see the relationship between the desired signal *S*_*σ*_ and the noise *N*_*σ*_ after averaging in the rectangular frame in Figure 5(d)).

Next, the right side of Equation (1) (i.e., the evaluation index for the scattering of the signal for the focusing operation) is obtained as shown in Figure 5(e). The maximum value (identified by a small circle) of all the calculated values on the right side was obtained via four repetitions (averaging operations) with the 300,000x image (Figure 5(a)) (the proper combination of improving SNR and reducing the number of pixels gives the maximum evaluation index value, as suggested in Equation (1) and Figure 4). Here, the number of pixels *M* is reduced to 40 × 30 (Figure 5(a′)). Similarly, size reductions to 80 × 40 for the 60,000x magnification and 20 × 15 for the 12,000x magnification are shown in Figures 5(b′) and 5(c′), respectively. Because the size reduction used for Figure 5(c) (12,000x) has no meaning (i.e., the very small black structures may not have been captured originally or may have already been removed), further discussion may not be necessary. Based on the results, Figure 5(a′) was selected as the appropriate image signal for the focusing operation. In this case (i.e., the use of 300,000x magnification and four repetitions), the right side of Equation (1) is improved by a factor of approximately four when compared with the unprocessed image with 60,000x magnification (Figure 5(b)), which may be suitable for fine focusing operations, if considered normally. When compared with Figure 2(b′), as mentioned previously, a very smooth graph for focusing operations should be obtained (not shown here).

Although the instrumental magnification was changed over a wide range from 12,000x to 300,000x in this experiment, the variation in the right side of Equation (1) observed in each graph is gradual. Similar improvements will thus be obtained over a specified wide range of magnifications.

### 2.5. Improvement of the Signal for Focusing Operations by Moving the Electron Beam

In the discussion leading up to the previous section, the acquired TV-scan SEM image was converted into a suitable signal for the focusing operation. Next, to improve the desired signal contained within the weak SEM signal fundamentally, the field of view with the maximum variation evaluation value is found actively near the observation region, as described in Reinforcement of the Signal for Focusing Based on the Active Image Processing Concept, by moving the electron beam. This experiment used the same SEM operating conditions and the same specimen that were used for Figure 5 and used the 300,000x magnification, as determined earlier. The search candidates are shown in Figures 3(a)–3(e) in order of their variation evaluation value, from large to small. We used the results in Figure 3(a) to achieve the purpose of the data processing step (there are smaller variations in a graph that was used for the focusing operation (not shown here)). Points a, b, c, d, and e in Figure 3(f) indicate the changes in the variation evaluation values that correspond to Figures 3(a)–3(e). Furthermore, for an overall view, all results obtained during the process of creation of Figure 3(c) using the SEM image in which priority is given to the SNR are shown (the numbers (0)–(5) in Figure 3 represent the numbers of repetitions of the averaging process). A graph is also presented in Figure 3(f) (incidentally, when compared with the graph obtained for the magnification of 300,000x shown in Figure 5(e), somewhat larger evaluation values were obtained in Figure 3(f) because of the differences in the fields of view). Based on the experimental results shown in Figures 5 and 3, we expect an improvement by several times in the stability of the signal used for the focusing operation.

### 2.6. Test of System Robustness against Noise during Fine Focusing for Extremely Noisy SEM Images

In this section, we demonstrate how powerful the improvements that have been discussed thus far are in terms of the stability of the signal used for focusing operations. These improvements are illustrated using the experimental results acquired under the near-minimum incident current condition in this study.  Figure 6(a) (300,000x magnification) shows an unprocessed TV-scan image acquired at 3 pA, which is similar to that acquired at 2 pA and shown in Figure 2(e) (60,000x). No fine details can be observed in this case because of the extremely severe noise. The signal used for focusing operations (graph in Figure 6(a′)) does not work at all under these conditions (Figures 6(a)–6(f) were acquired under the conditions at the right ends of graphs in Figures 6(a′)–6(f′), respectively, as indicated by the small circles). Unsurprisingly, when we use the SEM image acquired with priority given to the SNR (i.e., four repetitions of the averaging process, as described earlier), the signal for the focusing operations (Figure 6(b′)) works well, as expected. A focused (sharp) image corresponding to Figure 6(b′) and a defocused (blurred) image corresponding to Figure 6(c′) are shown in Figures 6(b) and 6(c), respectively. These images have identical views, and the graphs in Figures 6(b′) and 6(c′) are continuous (the graph in Figure 6(c′) shows results up to a point slightly further ahead in time). The bidirectional arrow in Figure 6(c′) indicates the difference between the two focal points as a difference in the SNR. We can see that the level of noise robustness of our system is sufficiently high, even under the incident current condition of approximately 3 pA, because this difference is much greater than the small variations spread throughout the signal.

To provide further stabilization, the moving average (i.e., the time average) of a graph for the focusing operation and the frame integration of the TV-scan images are also used in Figures 6(d)–6(f), although it is possible to perform focusing operations successfully using only the combination of methods proposed thus far. The principles of these general methods are not discussed here, but the former method produces averaged values that are calculated using only past data from the graph, while the latter requires old frames to acquire a noise-reduced image. Therefore, both methods introduce a time delay into the graph. To keep this delay to within a short time period, we experimentally select an average for four points on the graph and then integrate over four frames. When using these settings, the sensory time delay may then be within a suitable range for practical use, although the optimal combination has not been found to date. Almost perfectly stable results were obtained in the SEM operations of fine focusing (Figures 6(d) and 6(d′)) and astigmatism correction (Figures 6(e) and 6(e′)). The SEM operator only moved a *Y*-stigmator knob to correct the astigmatism. Both fine focusing and adjustment of the *X*-stigmator had already been completed (for fixed values). Here, the amplitude of the noise in Figures 6(d) and 6(e) is somewhat smaller than that observed in Figure 6(b) because of frame integration.

The improvement in the signal for focusing operations produced by moving the electron beam has not actually been used up to this point. To perform a practical operation similar to that for Figure 3, it is necessary to determine the best visual field under the condition that the fine focusing operation has not yet been achieved, as depicted in Figures 6(c) and 6(c′) (image blur, low SNR).  Figure 6(f′) shows a graph of the experimental results used to find a stronger signal (with higher SNR). In this experiment, we moved the electron beam rather than change the focus (the focus remains blurred). Despite the blurred image, we can easily distinguish between a visual field with a strong signal and a field with a weak signal in Figure 6(f′).  Figure 6(f) is blurred yet has higher contrast (i.e., a stronger signal) than the preceding images which were observed a few tens of seconds ago (not shown here). We expect better results when this visual field is used for fine focusing operations, although it may not be necessary for the current SEM conditions and specimen. This improvement in the signal may be required more for astigmatism correction than for fine focusing operations. In other words, the stigmator knob can be rotated relatively quickly under certain adverse conditions, because the change in the SNR may be relatively small. The focusing operation is then almost complete. If necessary, only the focal point will be finely readjusted within the field of view that the user actually wants to observe and acquire. To confirm the results of use of our method, Figure 6(g) shows a slow scan (80 s) SEM image that was acquired successfully at 60,000x magnification after adjustment at 300,000x. Under these very severe conditions (see Figures 2(e) and 6(a)), it is usually difficult for both experts and beginners to operate the SEM instrument well. In addition, using our method, Figure 6(h) (Au-coated silica particles) was acquired under conditions of slow scan (320 s), 100,000x magnification, 1 pA, and 15 kV. The resolution of the SEM used in this study is demonstrated fully.

Incidentally, the performance of our proposed method may be too high for use under normal SEM operating conditions, as shown in Figures 2(b) and 2(c). Under such operating conditions, we can expect the potential for use of shorter signal acquisition times for focusing operations, although we have not studied this specifically to date.

## 3. Conclusions

This study has demonstrated that the proposed techniques applied to improve the quality of a signal for use in fine focusing and astigmatism correction operations are effective for use in SEM. Our method for fundamental improvement of the signal for focusing operations works effectively, even under extremely noisy conditions. One notable advantage is that the focusing operation will have a very high probability of success, because the signal quality required for focusing is estimated in advance and the signal quality is then ensured using SEM and image processing techniques. This performance also demonstrates the potential of using a shorter signal acquisition time for focusing operations. This is an attractive prospect because it is usually difficult to reduce the number of data used without causing performance degradation. The parameters used in this study (e.g., 300,000x magnification) and the number of repetitions required (e.g., four averaging processes) were intended for a general-purpose SEM instrument and for use with a particular specimen. Depending on the differences among instruments in terms of their performance and required operating conditions, suitable parameters can be determined experimentally by finding the maximum value of the evaluation index (on the right side of Equation (1)). In future work, we may be able to use the results from this study to produce an automatic focusing device.

## Data Availability

The data used to support the findings of this study are available from the corresponding author upon reasonable request.
